# Supplementary material for: Identification of purine biosynthesis as an NADH-sensing pathway to mediate energy stress
Source: Nat Commun. 2022 Nov 17;13:7031. doi: 10.1038/s41467-022-34850-0 (PMC9672040; doi:10.1038/s41467-022-34850-0)
Supplement: Supplementary file 1 — Supplementary Information [file 41467_2022_34850_MOESM1_ESM.pdf]

Identification of purine biosynthesis as an NADH-sensing pathway to mediate energy stress

Yang et al.

Supplementary Figures

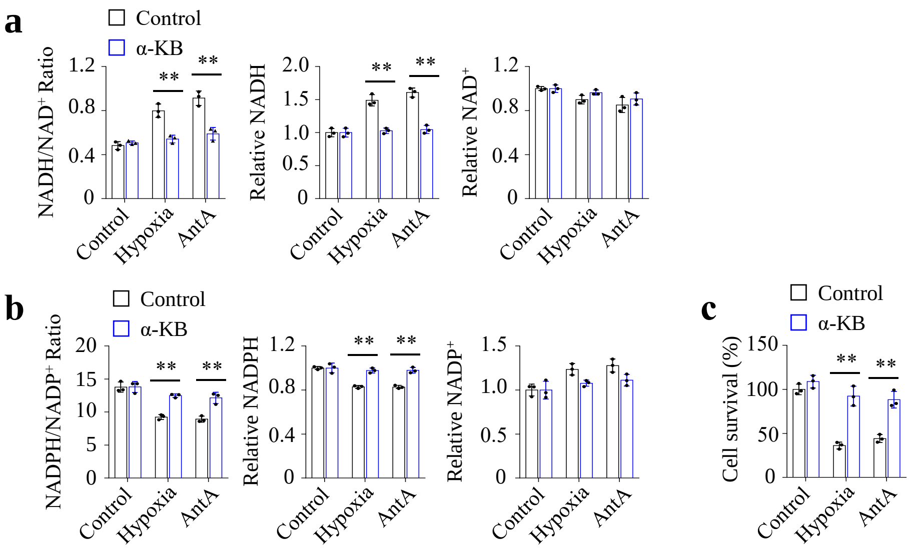

Supplementary Figure 1. Reductive stress induced by hypoxia and the inhibition of the electron transport chain. a,b, The NADH/NAD$^+$ ratio and NAD(H) content (**a**), and NADPH/NADP$^+$ ratio and NADP(H) content (**b**) in HeLa cells under hypoxia (0.5 % O_2) or ETC inhibition by antimycin A (1 μM). Cells were treated with or without α-ketobutyrate (α-KB, 2 mM) for 8 h. From left to right: for **a**, $**P = 0.00300$, $**P = 0.00307$, $**P = 0.00103$, $**P = 0.00046$, and for **b**, $**P = 0.00030$, $**P = 0.00448$, $**P = 0.00060$, $**P = 0.00094$. **c**, Cell survival of HeLa cells under hypoxia (0.5 % O_2) or ETC inhibition by antimycin A (1 μM). Cells were treated with or without α-KB (2 mM) for 72 h. From left to right: $**P = 0.00116$, $**P = 0.00187$. All data were from three biologically independent samples, and all experimental data were verified in at least two independent experiments. Error bars represent mean ± SD. $**P < 0.01$; Two-tailed Student's *t*-tests. Source data are provided as a Source Data file.

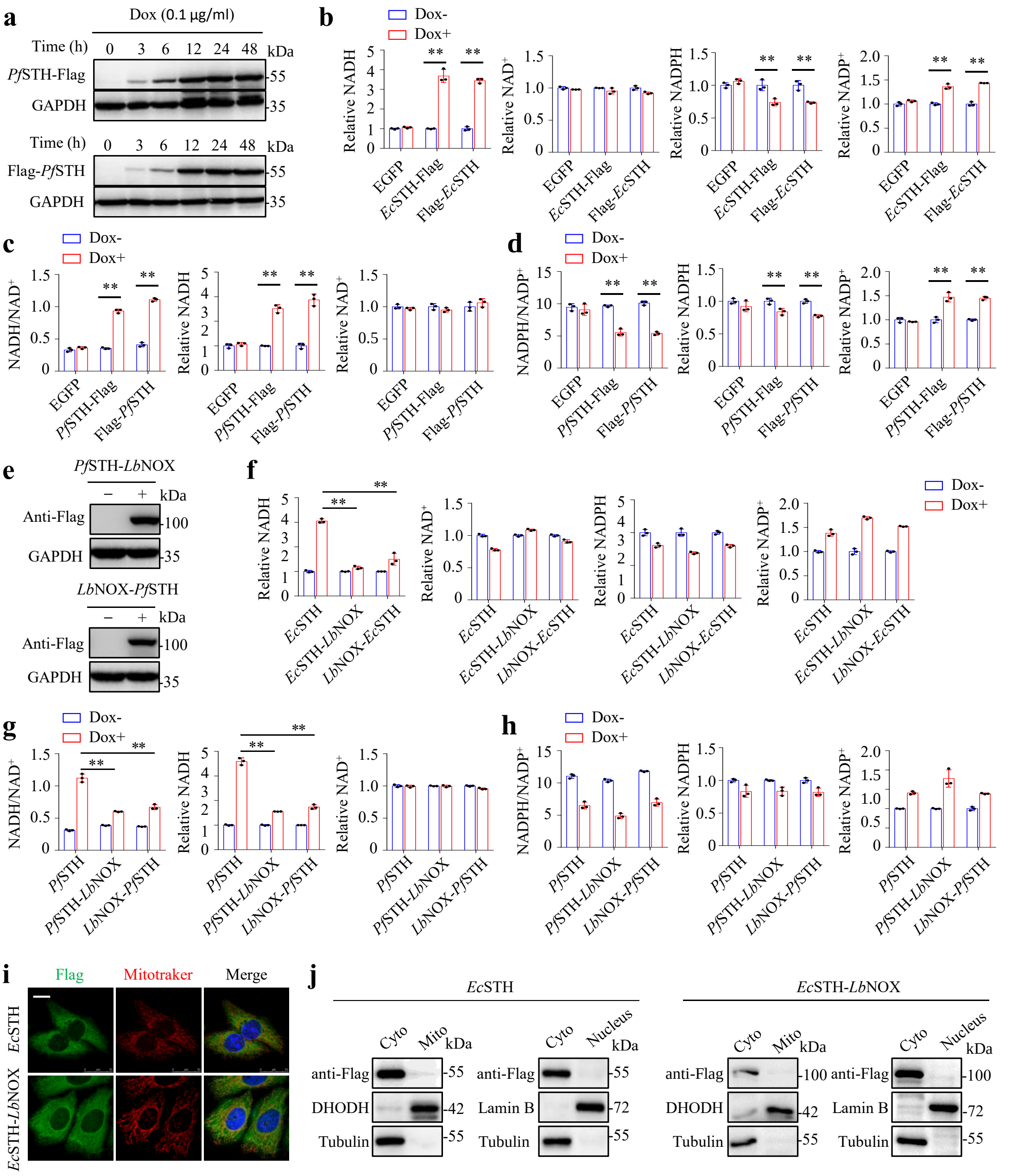

Supplementary Figure 2. Effects of STH and its fusion with *Lb*NOX on HeLa cells. a, Western blots for the expression of inducible Tet-on Flag-tagged *Pf*STH in HeLa cells induced by Dox (0.1 µg/ml) for different times. **b**, The relative levels of NADH, NAD^+, NADPH, and $NADP^+$ in HeLa cells expressing Tet-on Flag-tagged *Ec*STH cultured with Dox (0.1 µg/ml) for 12 h (n=3 biologically independent samples). From left to right: ***P* = 0.00148, ***P* = 1.19e-05, ***P* = 0.00946, ***P* = 0.00352, ***P* = 0.00046, ***P* = 5.68e-05. **c,d**, The ratios of cellular NADH/NAD^+ and NADPH/$NADP^+$ and the relative levels of NADH, NAD^+, NADPH, and $NADP^+$ in HeLa cells expressing Tet-on Flag-tagged *Pf*STH cultured with Dox (0.1 µg/ml) for 12 h (n=3 biologically independent samples). From left to right: for **c**, ***P* = 8.17e-06, ***P* = 8.00e-06, ***P* = 8.97e-05, ***P* = 0.00024, and for **d**, ***P* = 0.00021, ***P* = 3.72e-05, **P* = 0.01468, ***P* = 0.00042, ***P* = 0.00197, ***P* = 4.36e-05.**e**, Western blots for the expression of inducible Tet-on fusions of *Pf*STH with *Lb*NOX in HeLa cells induced by Dox (0.1 µg/ml) for

12 h. **f**, The relative levels of NADH, NAD^+, NADPH, and $NADP^+$ in HeLa cells expressing Tet-on fusion of *Ec*STH with *Lb*NOX cultured with Dox (0.1 μg/ml) for 12 h (n=3 biologically independent samples). From left to right: $**P$ = 6.60e-06, $**P$ = 0.00043. **g,h**, The ratios of cellular NADH/NAD^+ and NADPH/$NADP^+$ and the relative levels of NADH, NAD^+, NADPH, and $NADP^+$ in HeLa cells expressing Tet-on fusion of *Pf*STH with *Lb*NOX cultured with Dox (0.1 μg/ml) for 12 h (n=3 biologically independent samples). From left to right: $**P$ = 0.00014, $**P$ = 0.00046, $**P$ = 1.74e-05, $**P$ = 4.83e-05. **i,** The localization of *Ec*STH or *Ec*STH-*Lb*NOX in HeLa cells confirmed by immunofluorescence confocal microscope. Cells were induced by Dox (0.1 μg/ml) for 24 h. Scale bar, 10 μm. **j**, Cytoplasmic localization of *Ec*STH or *Ec*STH-*Lb*NOX in HeLa cells confirmed by Western blots for cellular fraction separation. All experimental data were verified in at least three independent experiments. Error bars represent mean ± SD. ** $P < 0.01$; Two-tailed Student's *t*-tests. Source data are provided as a Source Data file.

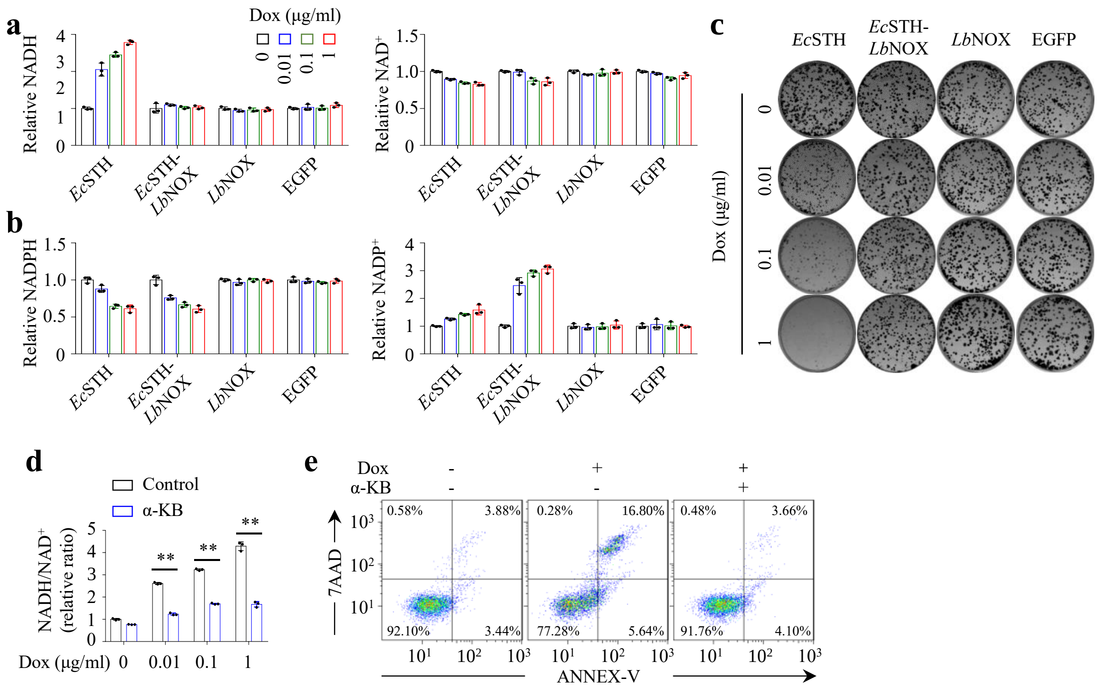

Supplementary Figure 3. Effects of *Ec*STH on the levels of NAD(P)H and NAD(P)$^+$ and colony formation ability in HeLa cells. a,b, The relative levels of NADH and NAD$^+$ (**a**), and NADPH and NADP$^+$ (**b**) in HeLa cells expressing inducible Tet-on *Ec*STH, *Ec*STH-*Lb*NOX, *Lb*NOX, or EGFP cultured with different concentrations of Dox for 24 h. **c,** Effects of EGFP, *Ec*STH, *Lb*NOX, or *Ec*STH-*Lb*NOX expression on colony formation in HeLa cells cultured with Dox, as indicated, for 10 days. **d**, The NADH/NAD$^+$ ratio in HeLa$^{\text{Tet-on } Ec\text{STH}}$ cells cultured with Dox for 12 h. Cells were treated with or without α-KB (2 mM). From left to right: $**P$ = 3.51e-06, $**P$ = 7.83e-07, $**P$ = 4.17e-05. **e**, Effects of α-KB treatment on cell apoptosis. Cell apoptosis was measured by Flow cytometry after treatment with Dox (1 μg/ml) and α-KB (2 mM) for 24 h. Data were from three biologically independent samples, and all experimental data were verified in at least three independent experiments. Error bars represent mean ± SD. $** P < 0.01$; Two-tailed Student's *t*-tests. Source data are provided as a Source Data file.

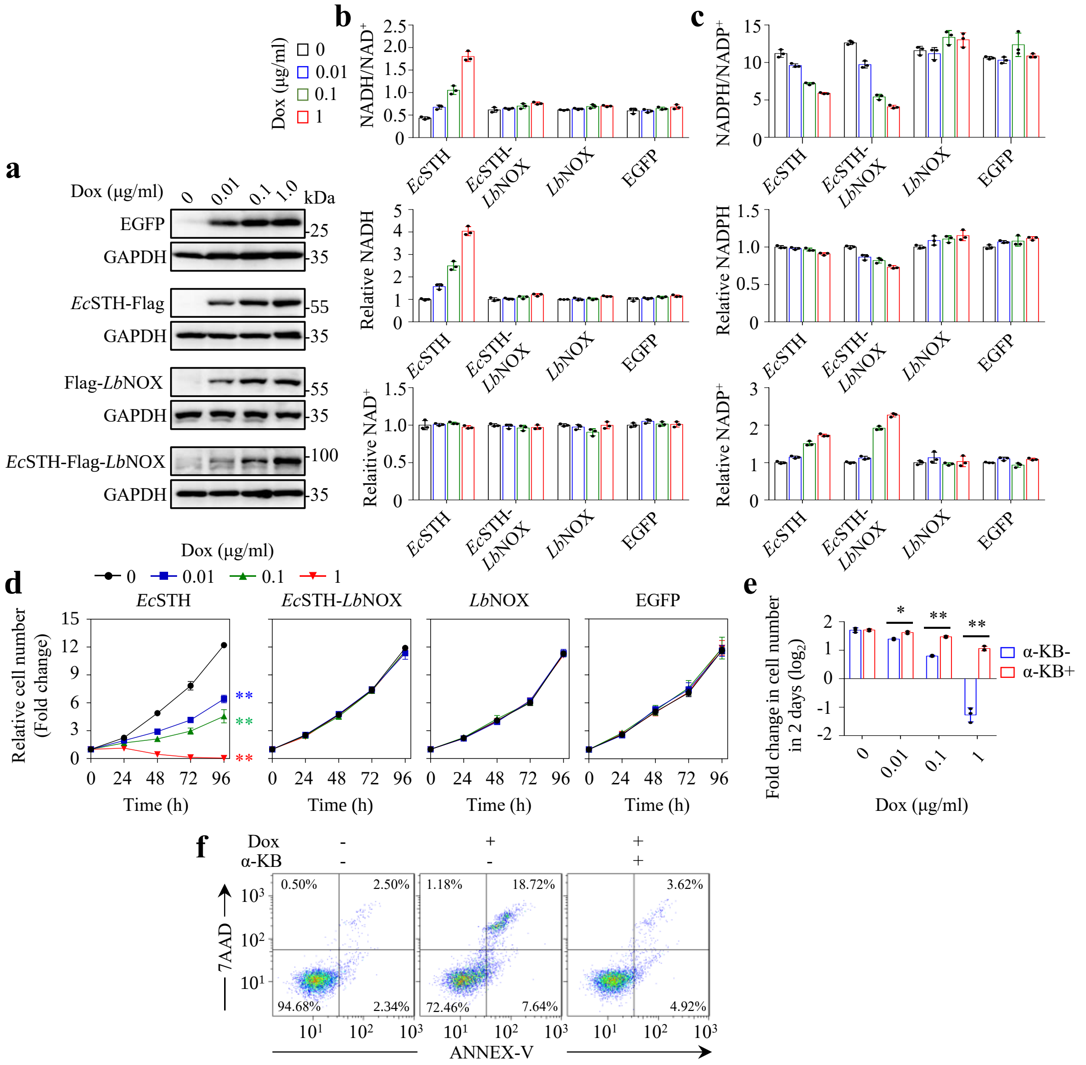

Supplementary Figure 4. Effects of *Ec*STH and its fusion with *Lb*NOX on MDA-MB-231 cells. a, Western blots for the expression of inducible Tet-on EGFP, *Ec*STH, *Lb*NOX, or *Ec*STH-*Lb*NOX in MDA-MB-231 cells induced by Dox for 12 h. **b,c,** The ratios of cellular NADH/NAD$^+$ (**b**) and NADPH/NADP$^+$ (**c**), and the relative levels of cellular NADH or NAD$^+$ (**b**), and NADPH or NADP$^+$ (**c**) in MDA-MB-231 cells expressing inducible Tet-on EGFP, *Ec*STH, *Lb*NOX, or *Ec*STH-*Lb*NOX cultured with different concentrations of Dox for 12 h (n=3 biologically independent samples). **d,** Effects of the expression of EGFP, *Ec*STH, *Lb*NOX or *Ec*STH-*Lb*NOX on cell growth in MDA-MB-231 cells cultured with different concentrations of Dox, as indicated (n=3 biologically independent samples). From top to bottom: $^{**}P < 0.0001$, $^{**}P < 0.0001$, $^{**}P < 0.0001$, two-way ANOVA. **e,** Effects of α-KB on the proliferation of MDA-MB-231$^{\text{Tet-on } EcSTH}$ cells. Cells were counted after treatment with Dox as indicated and α-KB (2 mM) for 48 h (n=3 biologically independent samples). From left to right: $^{**}P = 0.00201$, $^{**}P = 1.05\text{e-}05$, $^{**}P = 0.00010$, two-tailed Student's *t*-tests. **f,** Effects of α-KB treatment on cell apoptosis. Cell apoptosis was measured by Flow cytometry after treatment with Dox (1 μg/ml) and α-KB (2 mM) for 24 h. All experimental data were verified in at least three independent experiments. Error bars represent mean ± SD. ** $P < 0.01$. Source data are

provided as a Source Data file.

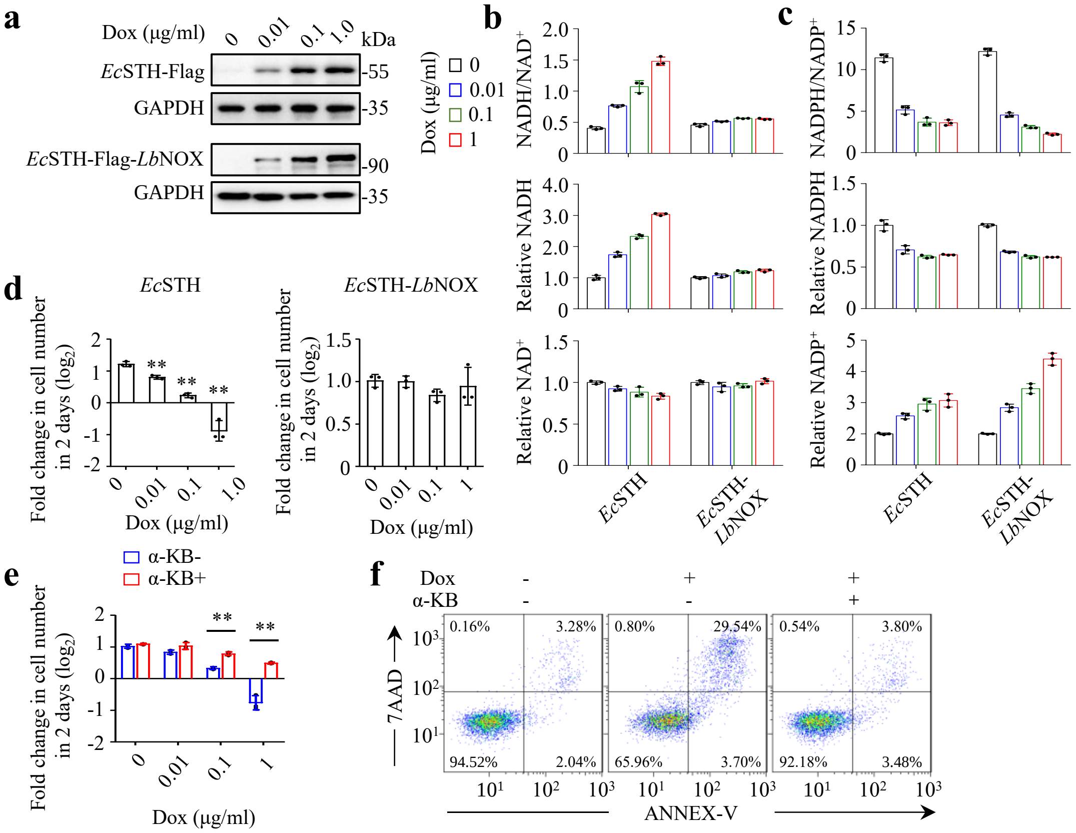

Supplementary Figure 5. Effects of *Ec*STH and its fusion with *Lb*NOX on MEF cells. a, Western blots for the expression of inducible Tet-on *Ec*STH or *Ec*STH-*Lb*NOX in MEF cells induced by Dox for 12 h. **b,c,** The ratios of cellular NADH/NAD^+ (**b**) and NADPH/$NADP^+$ (**c**), and the relative levels of cellular NADH or NAD^+ (**b**), and NADPH or $NADP^+$ (**c**) in MEF cells expressing inducible *Ec*STH or *Ec*STH-*Lb*NOX cultured with different concentrations of Dox for 12 h (n=3 biologically independent samples). **d,** Effects of the expression of *Ec*STH, or *Ec*STH-*Lb*NOX on cell growth in MEF cells cultured with different concentrations of Dox, as indicated (n=3 biologically independent samples). From left to right: $**P$ = 0.00198, $**P$ = 0.00010, $**P$ = 0.00038. **e,** Effects of α-KB on the proliferation of $MEF^{\text{Tet-on } EcSTH}$ cells. Cells were counted after treatment with Dox as indicated and α-KB (2 mM) for 48 h (n=3 biologically independent samples). From left to right: $**P$ = 0.00044, $**P$ = 0.00068. **f**, Effects of α-KB treatment on cell apoptosis. Cell apoptosis was measured by Flow cytometry after treatment with Dox (1 μg/ml) and α-KB (2 mM) for 24 h. All experimental data were verified in at least three independent experiments. Error bars represent mean ± SD. ** $P < 0.01$; two-tailed Student's *t*-tests. Source data are provided as a Source Data file.

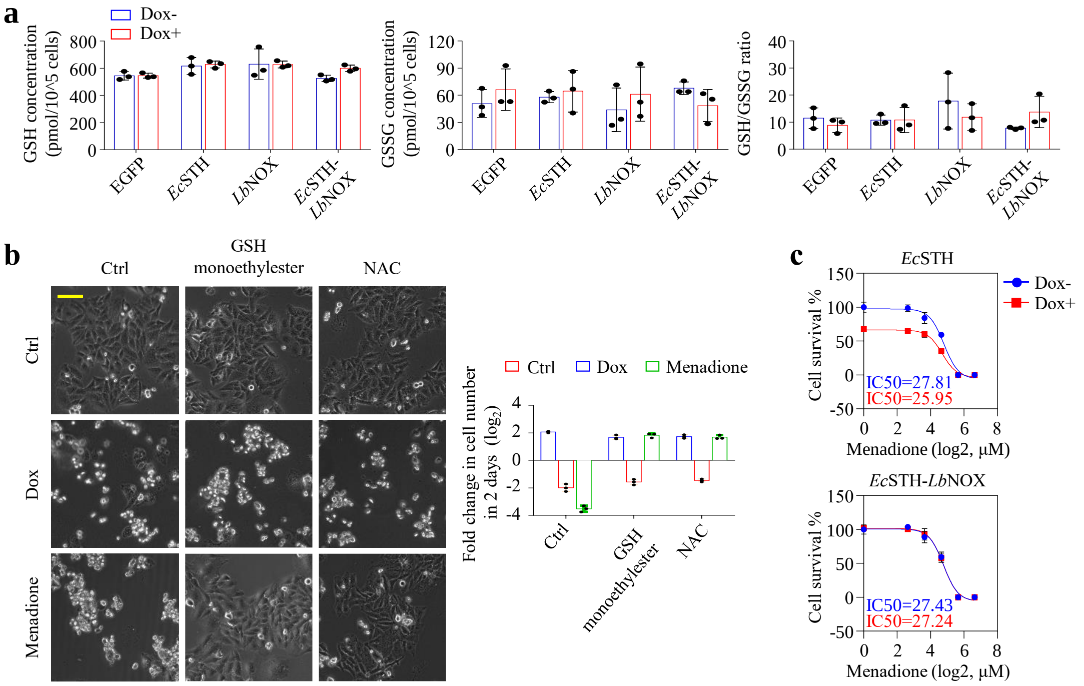

Supplementary Figure 6. *Ec*STH induced cell death is independent of oxidative stress. a, Cellular GSH and GSH/GSSG level in HeLa cells expressing inducible Tet-on EGFP, *Ec*STH, *Lb*NOX, or *Ec*STH-*Lb*NOX cultured with Dox (1 μg/ml) for 12 h. **b,** Effects of antioxidants on the proliferation of HeLa$^{\text{Tet-on }EcSTH}$ cells. Menadione treatment group as a positive control. Cells were counted after treatment with Dox (1 μg/ml) or menadione (50 μM) and antioxidants for 48 h. Glutathione monoethyl ester (0.5 mM), N-acetyl-L-cysteine (NAC, 5 mM). Scale bar, 10 μm. **c,** Sensitivity of HeLa$^{\text{Tet-on }EcSTH}$ or HeLa$^{\text{Tet-on }EcSTH\text{-}LbNOX}$ cells to menadione. Cells were pretreated with Dox (0.1 μg/ml) for 12 h to induce the expression of *Ec*STH or *Ec*STH-*Lb*NOX, and then exposed to menadione for 48 h. Data were from three biologically independent samples, and all experimental data were verified in at least two independent experiments. Error bars represent mean ± SD. Source data are provided as a Source Data file.

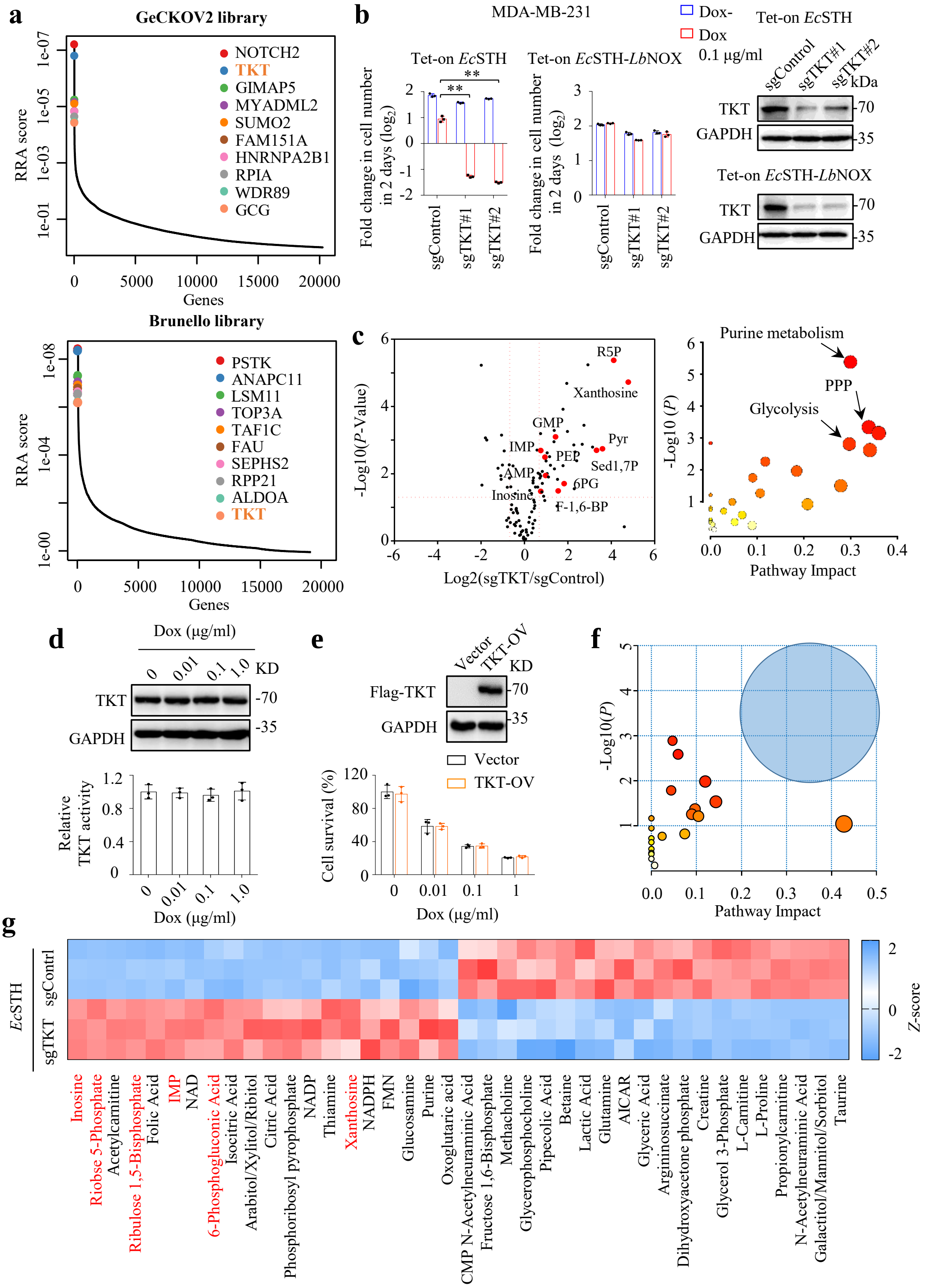

Supplementary Figure 7. Genome-wide negative CRISPR/Cas9 screening and metabolic profiling on NADH accumulation-associated pathways. a, Distribution of Robust Rank Aggregation (RRA) scores in genome-wide negative CRISPR/Cas9 screens. sgRNAs from both screens were ranked by their RRA score. The top 10 targeted genes were listed. **b,** Effects of TKT knockout on the proliferation of MDA-MB-231$^{\text{Tet-on } Ec\text{STH}}$ or MDA-MB-231$^{\text{Tet-on } Ec\text{STH-}Lb\text{NOX}}$ cells (n=3 biologically independent samples). Cells were counted after treatment with Dox (0.1

μg/ml) for 48 h. The immunoblot showed the levels of TKT. From left to right: $**P = 6.36e-06$, $**P = 3.33e-06$. **c,** Volcano plot (left) of the differential metabolites (two-tailed Student's *t*-tests) between sgTKT and sgControl in HeLa cells (n=3 biologically independent samples). The red highlights represent metabolites involved in purine metabolism, glycolysis, and the pentose phosphate pathway. KEGG pathway analysis (right) of the upregulated metabolites (fold change > 1.5, P value < 0.05) using MetaboAnalyst 5.0. **d,** Effects of *Ec*STH expression on TKT expression (top) and TKT activity (bottom) in $HeLa^{\text{Tet-on } \textit{Ec}\text{STH}}$ cells cultured with different concentrations of Dox for 24h as indicated (n=3 biologically independent samples). **e,** Effects of Flag-TKT overexpression on cell survival in $HeLa^{\text{Tet-on } \textit{Ec}\text{STH}}$ cells cultured with Dox, as indicated, for 48 h (n=3 biologically independent samples). **f,** KEGG Pathway analysis of the downregulated metabolites (fold change > 1.5, P value < 0.05) in $HeLa^{\text{Tet-on } \textit{Ec}\text{STH}}$ cells with sgTKT versus sgControl. **g,** Heatmap of the differential metabolites (Top 50) between sgTKT and sgControl in $HeLa^{\text{Tet-on } \textit{Ec}\text{STH}}$ cells. Z-score was calculated according to their fold changes (The abundance of metabolites was shown in Fig. 3f). **b,d,e,** Data were verified in at least two independent experiments. Error bars represent mean ± SD. ** $P < 0.01$; Two-tailed Student's *t*-tests. Source data are provided as a Source Data file.

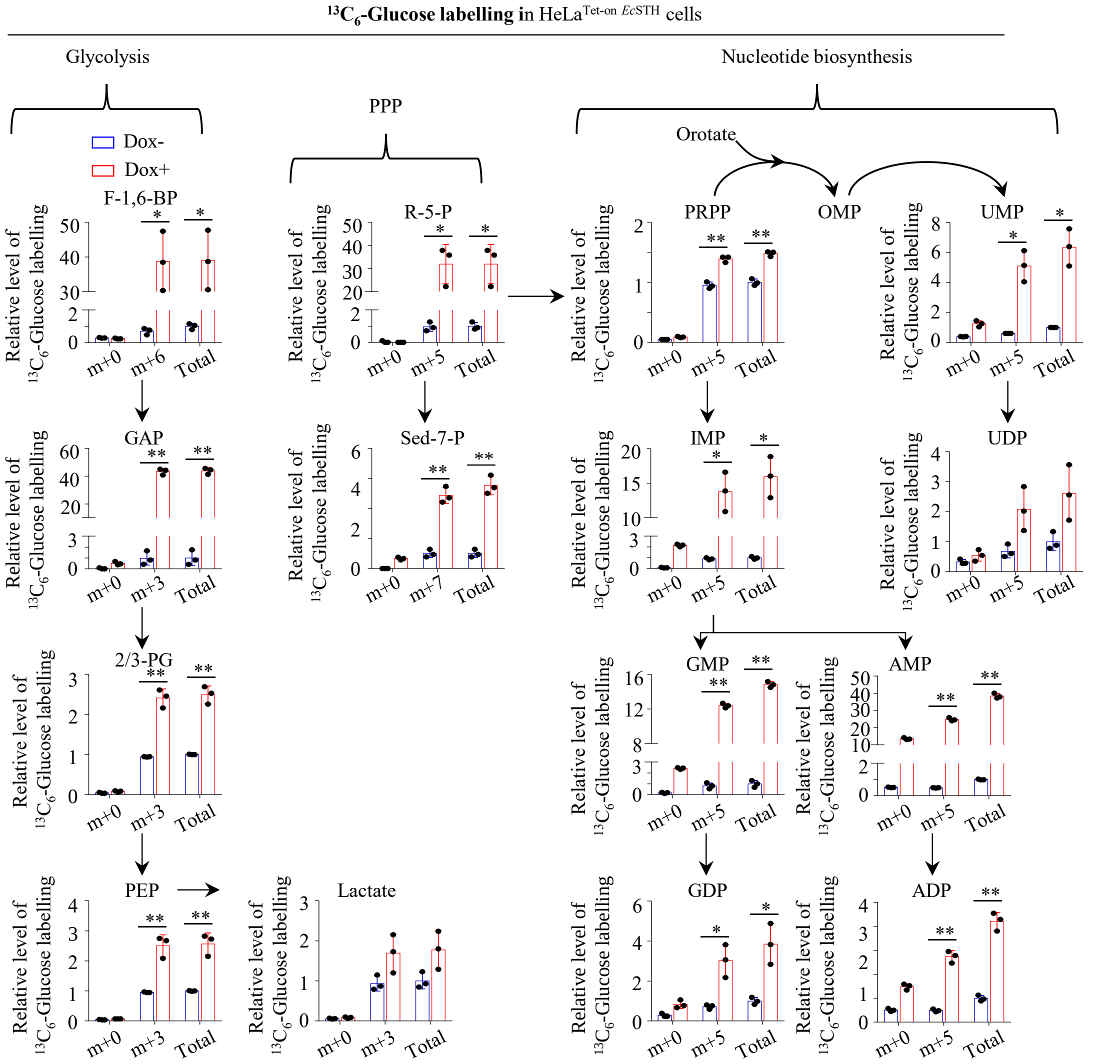

Supplementary Figure 8. Mass isotopomer analysis of cellular metabolites. Mass isotopomer analysis of metabolites in glycolysis, PPP, and nucleotide biosynthesis in HeLa$^{\text{Tet-on }Ec\text{STH}}$ cells cultured with ^{13}C-glucose (10 mM) for 8h (n=3 biologically independent samples). Cells were pre-induced by Dox (0.1 µg/ml) or water for 24 h. F-1,6-BP, fructose 1,6-diphosphate; GAP, glyceraldehyde 3 phosphate; 2/3-PG, 2/3-phosphoglyceric acid; PEP, phosphoenolpyruvate; R-5-P, ribose 5-phosphate; Sed-7-P, sedoheptulose 7-phosphate; PRPP, phosphoribosyl pyrophosphate. The scaling for the Y axes is normalized to the total level in the absence of Dox. From left to right: for F-1,6-BP, **P* = 0.01644, **P* = 0.01658; for GAP, ***P* = 5.80e-06, ***P* = 6.44e-06; for 2/3-PG, ***P* = 0.00038, ***P* = 0.00028; for PEP, ***P* = 0.00187, ***P* = 0.00172; for R-5-P, **P* = 0.02428, **P* = 0.02436; for Sed-7-P, ***P* = 0.00035, ***P* = 0.00030; for PRPP, ***P* = 0.00056, ***P* = 0.00029; for UMP, ***P* = 0.00166, ***P* = 0.00170; for IMP, **P* = 0.01581, **P* = 0.01283; for GMP, ***P* = 7.09e-07, ***P* = 7.66e-07; for AMP, ***P* = 1.56e-06, ***P* = 1.73e-06; for GDP, **P* = 0.03828, **P* = 0.03685; for ADP, ***P* = 0.00010, ***P* = 0.00014. Error bars represent mean ± SD. * $P < 0.05$, ** $P < 0.01$; Two-tailed Student's t-tests. Source data are provided as a Source Data file.

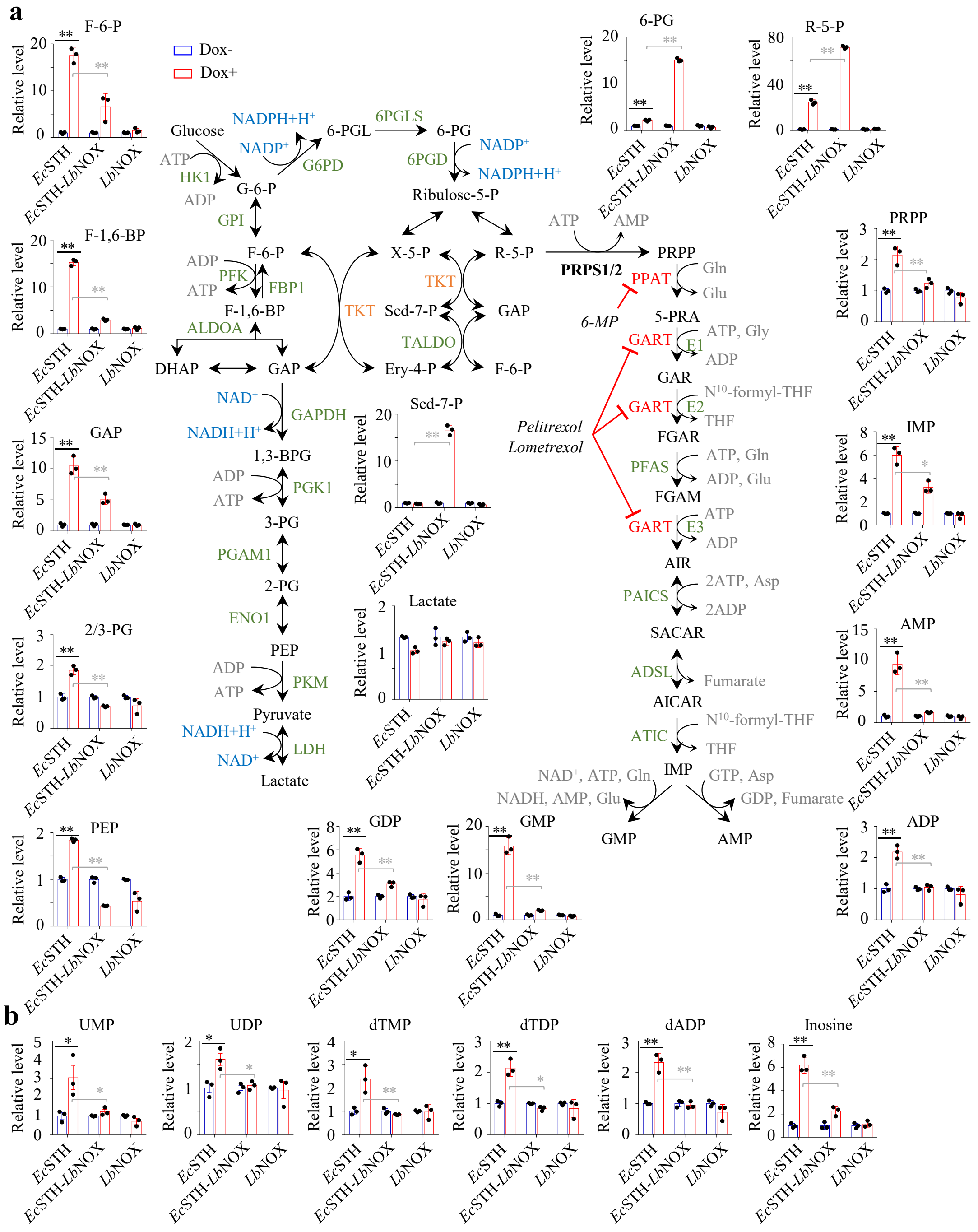

Supplementary Figure 9. Metabolomics analysis of cellular metabolites. a,b, Intracellular metabolite levels determined by LC-MS in HeLa cells expressing inducible Tet-on *Lb*NOX, *Ec*STH or *Ec*STH-*Lb*NOX cultured with Dox (0.1 μg/ml) for 24 h (n=3 biologically independent samples). From left to right: for F-6-P, $**P$ =5.65e-05, $**P$ = 0.00431; for F-1,6-BP, $**P$ = 3.77e-06, $**P$ = 8.22e-06; for GAP, $**P$ = 0.00039, $**P$ = 0.00536; for 2/3-PG, $**P$ = 0.00104, $**P$ = 0.00016; for PEP, $**P$ = 1.81e-05, $**P$ = 4.40e-07; for R-5-P, $**P$ = 3.49e-05, $**P$ = 4.04e-06; for Sed-7-P, $**P$ = 1.35e-05; for PRPP, $**P$ = 0.00267, $**P$ = 0.00843; for IMP, $**P$ = 0.00029, $**P$ = 0.00603; for GMP, $**P$ = 0.00015, $**P$ = 0.00019; for AMP, $**P$ = 0.00096, $**P$ = 0.00127; for GDP, $**P$ = 0.00081, $**P$ = 0.00258; for ADP, $**P$ = 0.00118, $**P$ = 0.00101; for UMP, $*P$ = 0.03513, $*P$ = 0.04720; for UDP, $*P$ = 0.01934, $*P$ = 0.01522;

for dTMP, *P = 0.01662, *P =0.01069; for dTDP, **P = 0.00221, **P = 0.00126; for dADP, **P = 0.00162, **P = 0.00171; for Inosine, **P = 0.00025, **P = 0.00108. Error bars represent mean ± SD. * $P < 0.05$, ** $P < 0.01$; Two-tailed Student's t-tests. Source data are provided as a Source Data file.

In the metabolic pathways in Panel (a), black letters indicate metabolites: G-6-P, glucose 6-phosphate; F-6-P, fructose 6-phosphate; F-1,6-BP, fructose 1,6-bisphosphate; DHAP, dihydroxyacetone phosphate; GAP, glyceraldehyde 3-phosphate; 1,3-BPG, 1,3-bisphosphoglycerate; 2/3-PG, 2/3-phosphoglycerate; PEP, phosphoenolpyruvate; 6-PGL, 6-phosphogluconolactone; 6-PG, 6-phosphogluconate; X-5-P, xylulose 5-phosphate; R-5-P, ribose 5-phosphate; Sed-7-P, sedoheptulose 7-phosphate; Ery-4-P, erythrose 4-phosphate; PRPP, phosphoribosyl pyrophosphate; 5-PRA, 5'-phosphoribosylamine; GAR, glycine amide ribonucleotide; FGAR, phosphoribosyl-*N*-formylglycineamide; FGAM, 5'-phosphoribosylformylglycinamidine; AIR, 5'-phosphoribosyl-5-aminoimidazole; SACAR, phosphoribosylaminoimidazolesuccinocarboxamide; AICAR, 5-aminoimidazole-4-carboxamide ribonucleotide; IMP, inosine monophosphate; GMP, guanosine monophosphate; AMP, adenosine monophosphate. Green letters represent enzymes: HK1, hexokinase 1; GPI, glucose phosphate isomerase; PFK, phosphofructokinase; FBP1, fructose-1,6-bisphosphatase 1; ALDOA, aldolase A; GAPDH, glyceraldehyde 3-phosphate; PGK1, phosphoglycerate kinase 1; PGAM1, phosphoglycerate mutase 1; ENO1, enolase 1; PKM, pyruvate kinase; LDH, lactate dehydrogenase; G6PD, glucose 6-phosphate dehydrogenase; PGLS, 6-phosphogluconolactonase; 6PGD, 6-phosphogluconate dehydrogenase; TKT, transketolase; TALDO, transaldolase; PRPS, ribose phosphate pyrophosphokinase; PPAT, amidophosphoribosyltransferase; GART, phosphoribosylglycinamide formyltransferase, phosphoribosylglycinamide synthetase and phosphoribosylaminoimidazole synthetase; PFAS, phosphoribosylformylglycinamidine synthase; PAICS, phosphoribosylaminoimidazole carboxylase and phosphoribosylaminoimidazolesuccinocarboxamide synthase; ADSL, adenylosuccinate lyase; ATIC, 5-amino-4-imidazole carboxamide ribonucleotide transformylase/IMP cyclohydrolase. Gray letters are the co-factors.

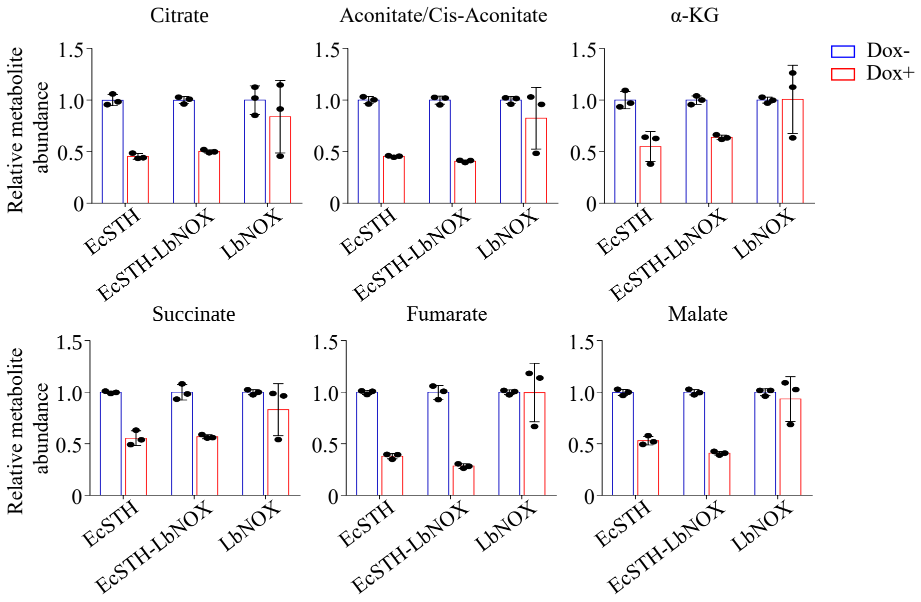

Supplementary Figure 10. The cellular levels of TCA cycle metabolites. Intracellular metabolite levels determined by LC-MS in HeLa cells expressing inducible Tet-on *Lb*NOX, *Ec*STH or *Ec*STH-*Lb*NOX cultured with Dox (0.1 μg/ml) for 24 h (n=3 biologically independent samples). Error bars represent mean ± SD. Source data are provided as a Source Data file.

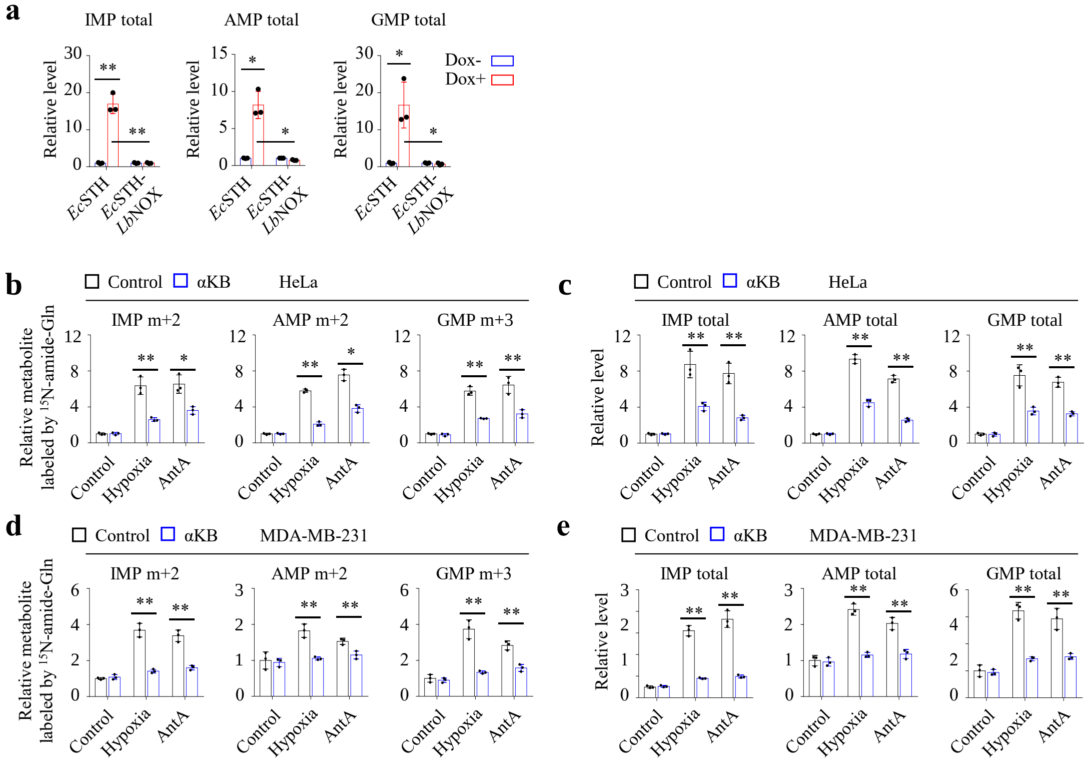

Supplementary Figure 11. NADH accumulation induced by hypoxia or ETC inhibition promotes purine biosynthesis. a, The total level of IMP, AMP and GMP in HeLaTet-on EcSTH and HeLaTet-on EcSTH-LbNOX cells cultured with glutamine-15N-amide (1 mM) for 12 h. Cells were pretreated with Dox (0.1 μg/ml) for 12 h. From left to right: $**P = 0.00046$, $**P = 0.00046$, $*P = 0.02108$, $*P = 0.01958$, $*P = 0.04813$, $*P = 0.04660$. **b-e,** The labeled fraction (**b,d**) or the total level (**c,e**) of IMP, AMP and GMP in HeLa cells (**b,c**) or MDA-MB-231 cells (**d,e**) cultured with 1 mM of glutamine-^{15}N-amide under hypoxia (0.5% O_2) or ETC inhibition by antimycin A (1 μM) for 12 h. From left to right: for **b**, $**P = 0.00282$, $*P = 0.01008$, $**P = 3.28e\text{-}05$, $*P = 0.01008$, $**P = 0.00036$, $**P = 0.00606$; for **c**, $**P = 0.00645$, $**P = 0.00191$, $**P = 0.00019$, $**P = 5.64e\text{-}05$, $**P = 0.00538$, $**P = 0.00053$; for **d**, $**P = 0.00056$, $**P = 0.00089$, $**P = 0.00217$, $**P = 0.00938$, $**P = 0.00141$, $**P = 0.00241$; for **e**, $**P = 9.13e\text{-}05$, $**P = 0.00031$, $**P = 0.00021$, $**P = 0.00231$, $**P = 0.00064$, $**P = 0.00400$. Data were from three biologically independent samples. Error bars represent mean ± SD. $*P < 0.05$, $**P < 0.01$; Two-tailed Student's *t*-tests. Source data are provided as a Source Data file.

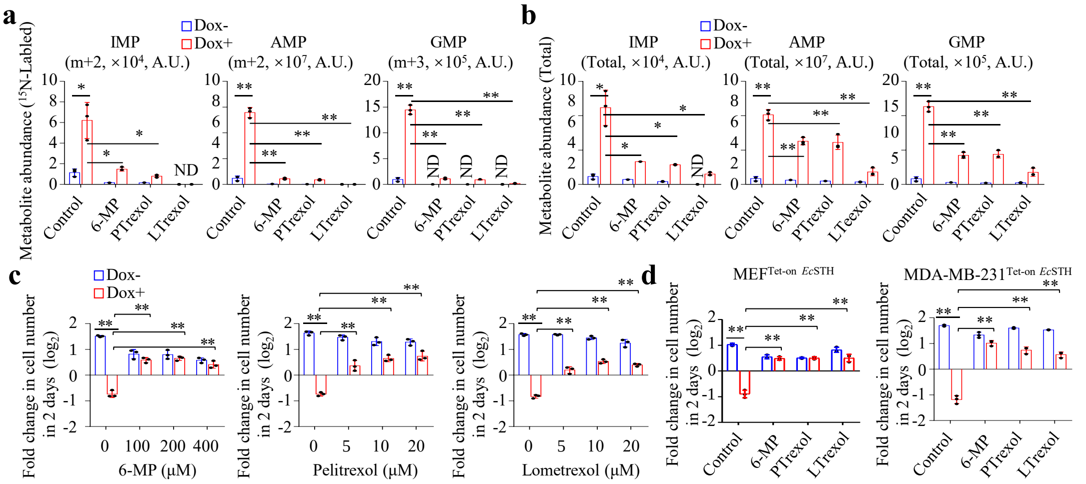

Supplementary Figure 12. *Ec*STH-induced cell death is triggered by dysregulated purine biosynthesis. a,b, The labeled fraction (**a**) or the total level (**b**) of IMP, AMP, and GMP in HeLa $^{\text{Tet-on } Ec\text{STH}}$ cells cultured with glutamine-^{15}N-amide (1 mM) for 12 h (n=3 biologically independent samples). Cells were pretreated with Dox (0.1 μg/ml) for 12 h to induce the expression of *Ec*STH. 6-MP (300 μM), PTrexol (10 μM), LTrexol (10 μM). From left to right: for IMP (**a**), **P* = 0.03328, **P* = 0.04183, **P* = 0.03293; for AMP (**a**), ***P* = 3.52e-05, ***P* = 2.41e-05, ***P* = 2.21e-05, ***P* = 1.66e-05; for GMP (**a**), ***P* = 1.78e-05, ***P* = 1.45e-05, ***P* = 1.31e-05, ***P* = 1.05e-05; for IMP (**b**), **P* = 0.01940, **P* = 0.03385, **P* = 0.03031, **P* = 0.02195; for AMP (**b**), ***P* = 3.94e-05, ***P* = 0.00193, ***P* = 0.00550, ***P* = 0.00010; for GMP (**a**), ***P* = 2.41e-05, ***P* = 0.00014, ***P* = 0.00020, ***P* = 6.41e-05. **c,** Effects of purine biosynthesis inhibitors treatment on cell survival in HeLa$^{\text{Tet-on } Ec\text{STH}}$ cells. Cells were pretreated with the indicated inhibitors for 2 h and counted after treatment with Dox (1 μg/ml) and inhibitors for 48 h (n=3 biologically independent samples). From left to right: for 6-MP, ***P* = 9.67e-05, ***P* = 0.00017, ***P* = 0.00010, ***P* = 0.00052; for Pelitrexol, ***P* = 2.95e-05, ***P* = 0.00094, ***P* = 7.85e-05, ***P* = 0.00020; for Lometrexol, ***P* = 8.25e-05, ***P* = 0.00028, ***P* = 2.30e-05, ***P* = 1.67e-05. **d,** Effects of purine biosynthesis inhibitors on proliferation of MEF$^{\text{Tet-on } Ec\text{STH}}$ or MDA-MB-231$^{\text{Tet-on } Ec\text{STH}}$ cells (n=3 biologically independent samples). Cells were pretreated with the indicated inhibitors for 2 h and counted after treatment with Dox (1 μg/ml) and inhibitors for 48 h. From left to right: for MEF, ***P* = 3.14e-05, ***P* = 0.00013, ***P* = 0.00011, ***P* = 0.00030; for MDA-MB-231, ***P* = 6.09e-06, ***P* = 3.42e-05, ***P* = 8.21e-05, ***P* = 9.36e-05. 6-MP (300 μM), PTrexol (10 μM), LTrexol (10 μM). **a,b,** Data were verified in at least three independent experiments. Error bars represent mean ± SD. * $P < 0.05$, ** $P < 0.01$; Two-tailed Student's *t*-tests. Source data are provided as a Source Data file.

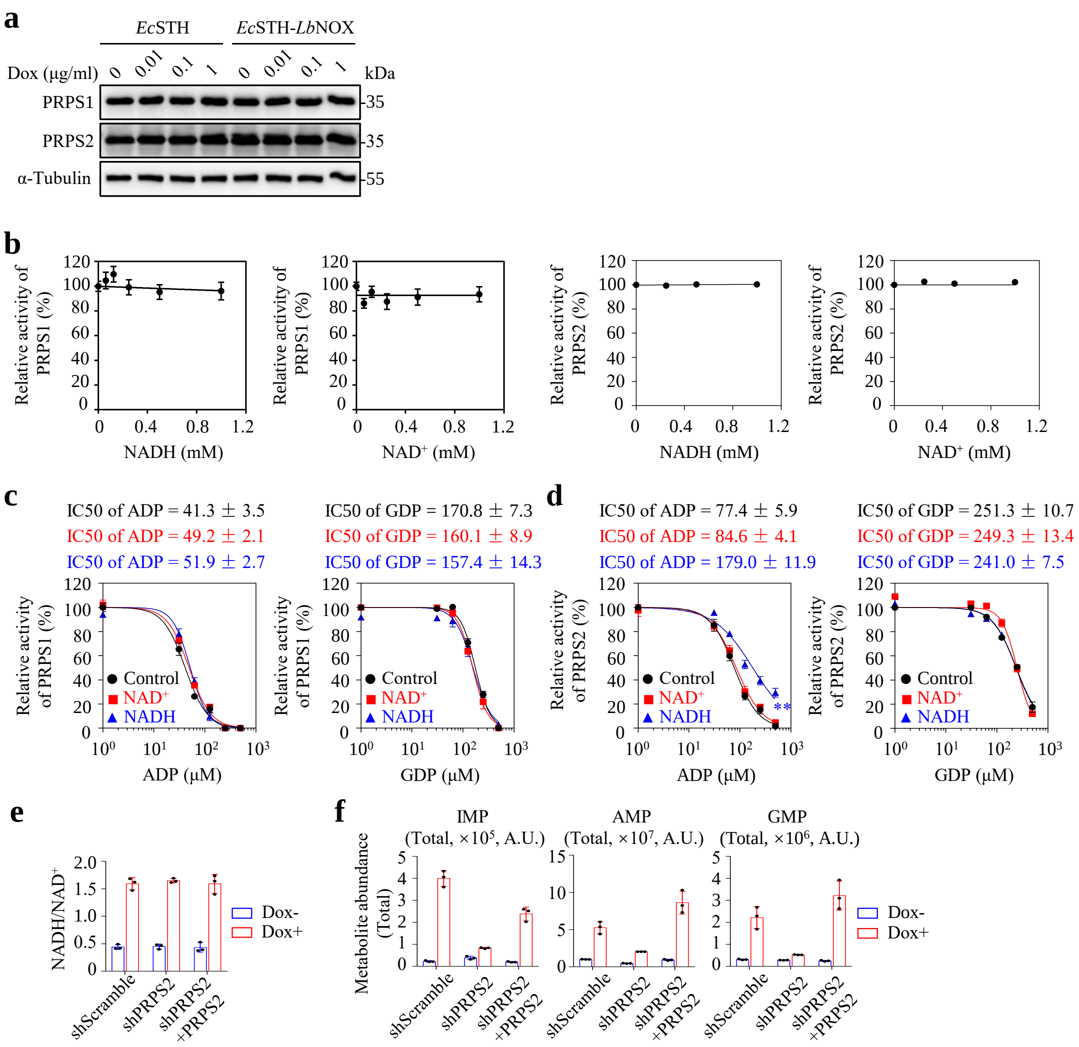

Supplementary Figure 13. Effects of NADH and NAD^+ on the activities of PRPS1/2. a, The expression of PRPS1 and PRPS2 in HeLa$^{\text{Tet-on }\textit{Ec}\text{STH}}$ and HeLa$^{\text{Tet-on }\textit{Ec}\text{STH-}\textit{Lb}\text{NOX}}$ cells cultured with Dox as indicted for 24 h. **b,** The effects of NAD or NADH on PRPS1 or PRPS2 activity (n=3 biologically independent samples). **c,d,** The effects of NAD (200 μM) or NADH (200 μM) on PRPS1 or PRPS2 activity in the presence of different concentrations of ADP or GDP, as indicated (n=3 biologically independent samples). ***P* < 0.0001, two-way ANOVA. **e,** Effect of PRPS2 knockdown or rescue on NADH/NAD^+ in HeLa$^{\text{Tet-on }\textit{Ec}\text{STH}}$ cells cultured with Dox (1 μg/ml) for 12 h (n=3 biologically independent samples). **f,** The total abundance of IMP, AMP, and GMP in PRPS2 knockdown as well as PRPS2 rescue HeLa$^{\text{Tet-on }\textit{Ec}\text{STH}}$ cells cultured with glutamine-^{15}N-amide (1 mM) for 12 h (n=3 biologically independent samples). Cells were pretreated with Dox (0.1 μg/ml) for 12 h. **a-e,** Data were verified in at least two independent experiments. Error bars represent mean ± SD. ** *P* < 0.01. Source data are provided as a Source Data file.

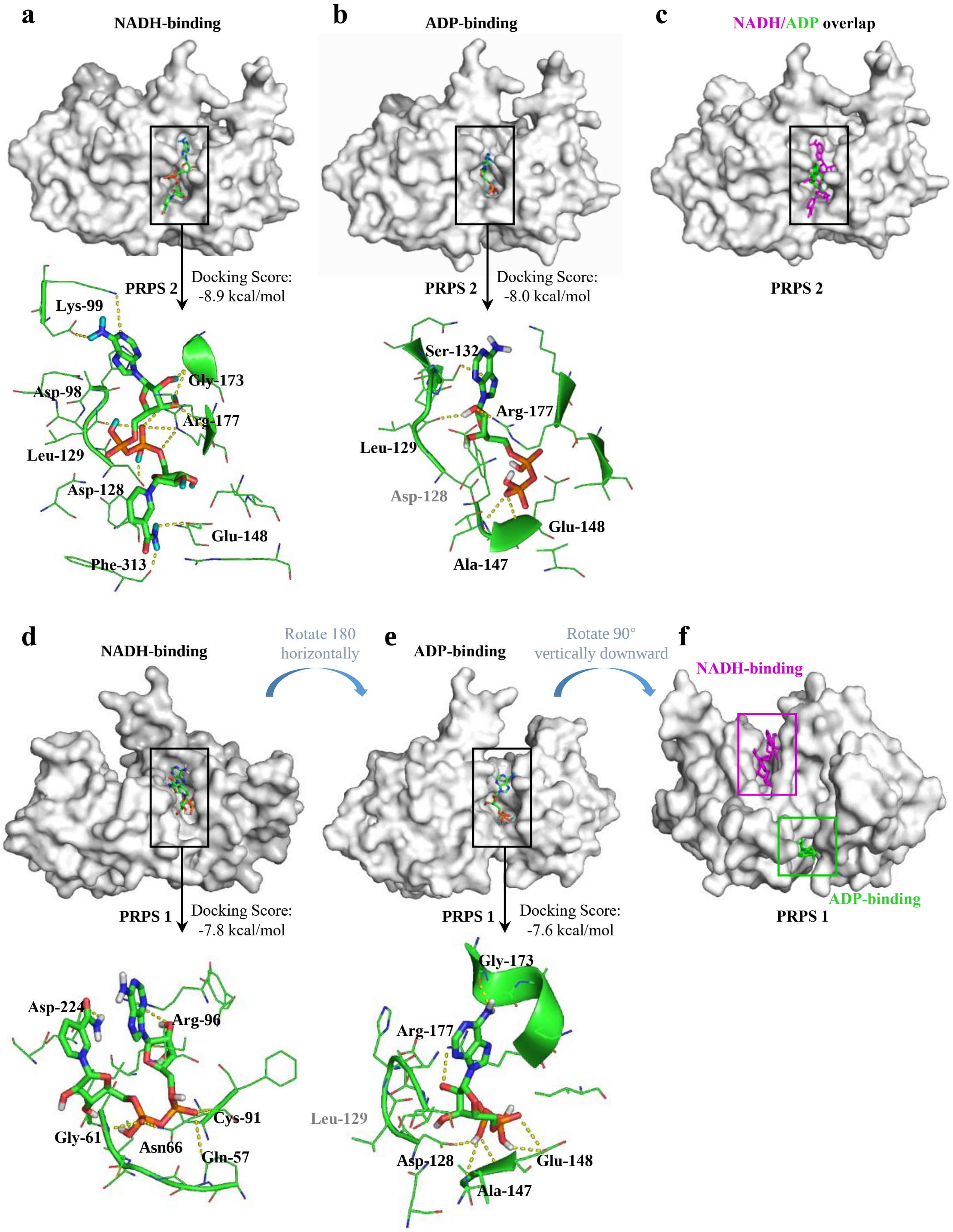

Supplementary Figure 14. The molecular docking of NADH and ADP to PRPS1/2. a-c, The molecular docking of NADH and ADP to PRPS2. NADH-binding site in PRPS2 (**a**), ADP-binding site in PRPS2 (**b**), Merged (**c**). **d-f,** The molecular docking of NADH and ADP to PRPS1. NADH-binding site in PRPS1 (**a**), ADP-binding site in PRPS1 (**b**), Merged (**c**).

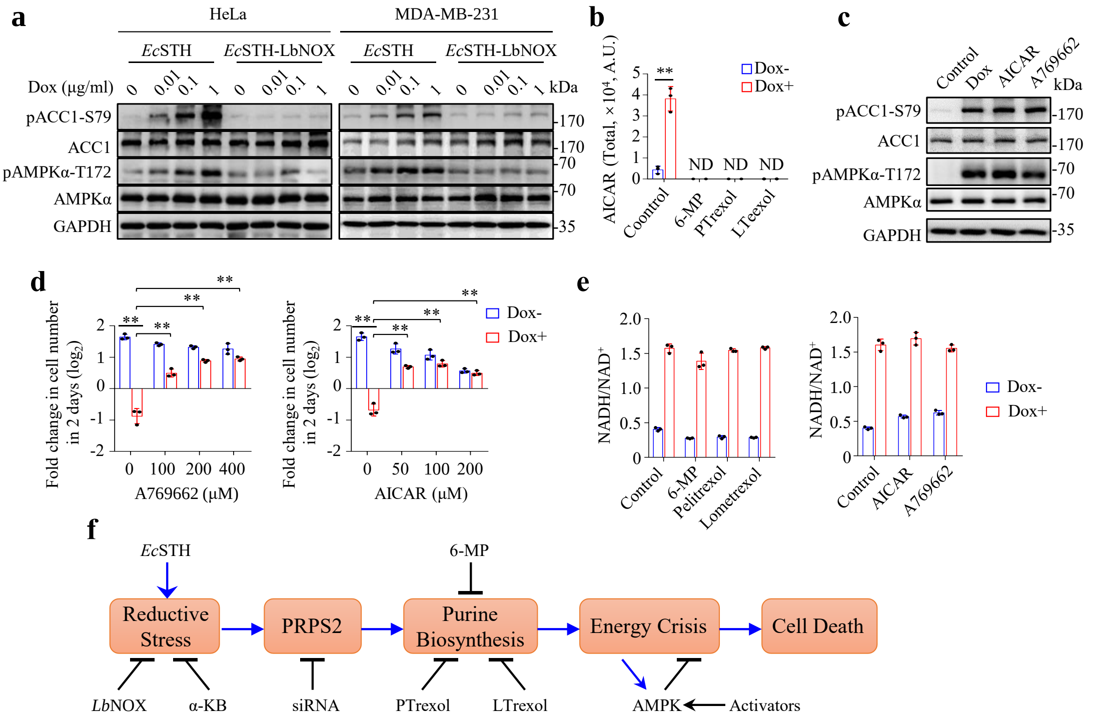

Supplementary Figure 15. *Ec*STH-induced NADH accumulation promotes cell death through energy stress. a, Immunoblots showing the effects of *Ec*STH or *Ec*STH-*Lb*NOX expression on AMPK pathway in HeLa and MDA-MB-231 cells cultured with Dox (0, 0.01, 0.1, 1 μg/ml) for 12 h. **b**, The abundance of AICAR in HeLa$^{\text{Tet-on } Ec\text{STH}}$ cells cultured with Dox (0.1 μg/ml) for 24 h (n=3 biologically independent samples). 6-MP (300 μM), PTrexol (10 μM), LTrexol (10 μM). $^{**}P = 2.30\text{e-}05$. **c,** Immunoblots showing the effects of Dox (0.1 μg/ml) and AMPK activators, AICAR (400 μM) and A769662 (100 μM), on AMPK pathway in HeLa$^{\text{Tet-on } Ec\text{STH}}$ cells cultured without Dox for 48 h. **d**, Effects of AMPK activators on the proliferation of HeLa$^{\text{Tet-on } Ec\text{STH}}$ cells cultured with Dox (1 μg/ml) for 48 h (n=3 biologically independent samples). Cells were pretreated with the indicated drugs for 2 h before Dox addition. From left to right: for A769662, $^{**}P = 7.27\text{e-}05$, $^{**}P = 0.00098$, $^{**}P = 0.00025$, $^{**}P = 0.00023$; for AICAR, $^{**}P = 5.47\text{e-}05$, $^{**}P = 0.00025$, $^{**}P = 0.00029$, $^{**}P = 0.00057$. **e** Effects of purine synthesis inhibitors or AMPK activators on cellular NADH/NAD$^+$ ratio in HeLa$^{\text{Tet-on } Ec\text{STH}}$ cells cultured with Dox (1 μg/ml) for 12 h (n=3 biologically independent samples). Cells were pretreated with the indicated drugs for 2 h before Dox addition. 6-MP (300 μM), PTrexol (10 μM), LTrexol (10 μM), AICAR (400 μM) and A769662 (100 μM). **f,** The working model for the axis of "Reductive stress-deregulated purine biosynthesis-energy stress". **a,c-e,** Data were verified in at least three independent experiments. Error bars represent mean ± SD. ** $P < 0.01$; Two-tailed Student's t-tests. Source data are provided as a Source Data file.

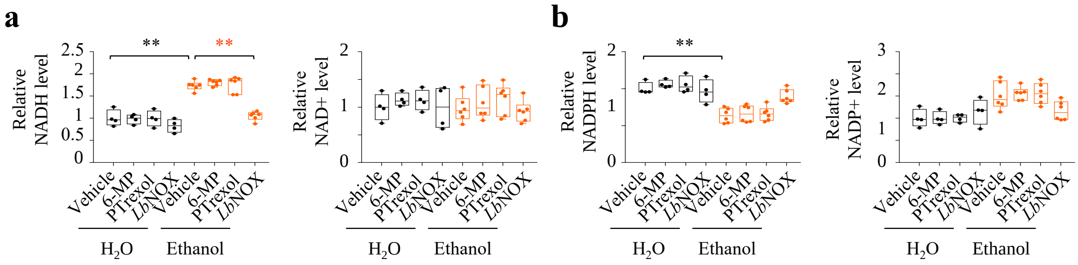

Supplementary Figure 16. Ethanol administration-induced reductive. a,b, Effects of acute ethanol gavage on the content of NADH, NAD^+, NADPH, or $NADP^+$ in liver tissue. From left to right: $**P = 4.05e\text{-}05$, $**P = 4.60e\text{-}07$, $**P = 0.00031$. H_2O group (n = 4 biologically independent animals) and ethanol group (n=6 biologically independent animals). Boxes represent minima to maxima (line at median) with whiskers at 1.5*IQR. ** $P < 0.01$; Two-tailed Student's *t*-tests. Source data are provided as a Source Data file.

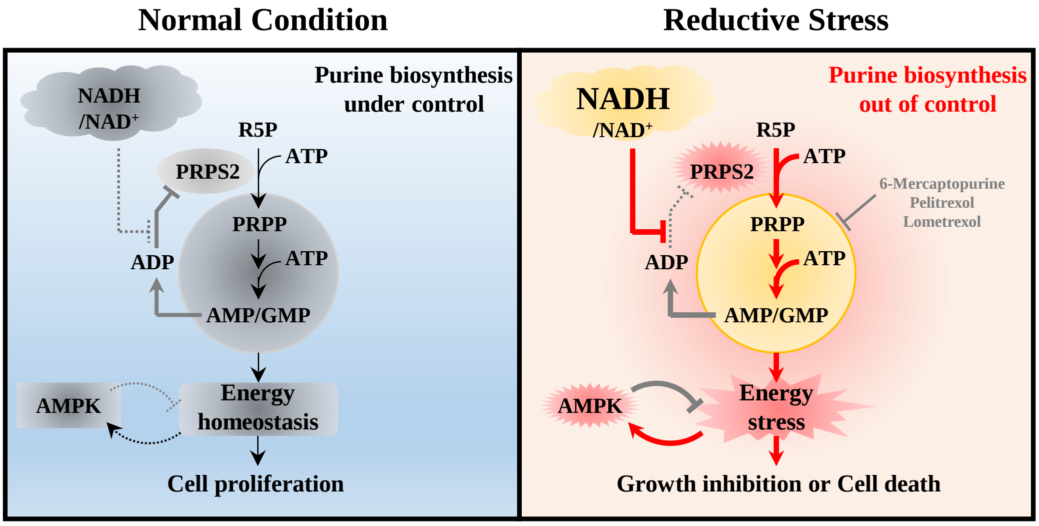

Supplementary Figure 17. The working model for the regulation of purine biosynthesis by reductive stress. Under normal conditions, NADH, coupled with ATP generation, promotes purine biosynthesis that is strictly regulated by the end product ADP, thus cells can maintain the homeostasis of cellular energy metabolism. When cells suffer from reductive stress, such as hypoxia and mitochondrial dysfunction, the resultantly accumulated NADH, uncoupled with ATP generation, relieves the inhibition of PRPS2 afforded by ADP, and thus deregulates purine biosynthesis to promote energy stress.
