# Supplementary material for: Identification of purine biosynthesis as an NADH-sensing pathway to mediate energy stress
Source: Nat Commun. 2022 Nov 17;13:7031. doi: 10.1038/s41467-022-34850-0 (PMC9672040; doi:10.1038/s41467-022-34850-0)
Supplement: Supplementary file 3 — Description of Additional Supplementary Files [file 41467_2022_34850_MOESM3_ESM.pdf]

File Name: Supplementary Data 1
Description: Distribution of Robust Rank Aggregation (RRA) scores in genome-wide negative CRISPR/Cas9 screens (GeCKOV2 library and Brunello library);

File Name: Supplementary Data 2
Description: Targeted metabonomics unprocessed data determined by LC-MS in HeLa cells expressing inducible Tet-on *Lb*NOX, *Ec*STH or *Ec*STH-*Lb*NOX cultured with Dox (0.1 μg/ml) for 24 h.
