# Supplementary material for: Identification of purine biosynthesis as an NADH-sensing pathway to mediate energy stress
Source: Nat Commun. 2022 Nov 17;13:7031. doi: 10.1038/s41467-022-34850-0 (PMC9672040; doi:10.1038/s41467-022-34850-0)
Supplement: Supplementary file 6 — Reporting Summary [file 41467_2022_34850_MOESM6_ESM.pdf]

nature portfolio

Corresponding author(s): Binghui Li

Last updated by author(s): Nov 8, 2022

Reporting Summary

Nature Portfolio wishes to improve the reproducibility of the work that we publish. This form provides structure for consistency and transparency in reporting. For further information on Nature Portfolio policies, see our Editorial Policies and the Editorial Policy Checklist.

Statistics

For all statistical analyses, confirm that the following items are present in the figure legend, table legend, main text, or Methods section.

n/a	Confirmed	
☐	☒	The exact sample size (*n*) for each experimental group/condition, given as a discrete number and unit of measurement
☐	☒	A statement on whether measurements were taken from distinct samples or whether the same sample was measured repeatedly
☐	☒	The statistical test(s) used AND whether they are one- or two-sided<br>*Only common tests should be described solely by name; describe more complex techniques in the Methods section.*
☒	☐	A description of all covariates tested
☒	☐	A description of any assumptions or corrections, such as tests of normality and adjustment for multiple comparisons
☐	☒	A full description of the statistical parameters including central tendency (e.g. means) or other basic estimates (e.g. regression coefficient) AND variation (e.g. standard deviation) or associated estimates of uncertainty (e.g. confidence intervals)
☐	☒	For null hypothesis testing, the test statistic (e.g. *F*, *t*, *r*) with confidence intervals, effect sizes, degrees of freedom and *P* value noted<br>*Give P values as exact values whenever suitable.*
☒	☐	For Bayesian analysis, information on the choice of priors and Markov chain Monte Carlo settings
☒	☐	For hierarchical and complex designs, identification of the appropriate level for tests and full reporting of outcomes
☒	☐	Estimates of effect sizes (e.g. Cohen's *d*, Pearson's *r*), indicating how they were calculated

Our web collection on statistics for biologists contains articles on many of the points above.

Software and code

Policy information about availability of computer code

Data collection	TriStar² LB 942 multimode microplate reader (Berthold Technologies) was used to collect luminescence, fluorescent and absorption data; Deep sequencing was performed by HiSeq-PE250; Metabolomic data was performed using TSQ Quantiva triple quadrupole mass spectrometer coupled to a Dionex Ulti-Mate 3000 UPLC system (Thermo Fisher); Cell number and cell apoptosis rate were measured by Muse Cell Analyzer (Luminex Cooperation).ChemBio 3D software was used to calculate and derive the 3D structure by minimizing energy. PyMOL 2.4.0 software was used to perform the dehydration of the receptor protein. Autodock software was used to carry out hydrogenation and charge calculation of proteins.Autodock Vina was used to dock the receptor protein with the small molecule ligands.SPR analysis was conducted with a Biacore T200 biomolecular interaction analysis system (GE Healthcare).
Data analysis	GraphPad PRISM 7.0 were used for bar graphs output and statistic analysis. MetaboAnalyst 5.0 were used for metabolomics data analysis. Significantly enriched or depleted sgRNAs were identified using MAGeCK 0.5.9. Cell apoptosis rate was analyzed by FlowJo 7.6.

For manuscripts utilizing custom algorithms or software that are central to the research but not yet described in published literature, software must be made available to editors and reviewers. We strongly encourage code deposition in a community repository (e.g. GitHub). See the Nature Portfolio guidelines for submitting code & software for further information.

Data

Policy information about availability of data

All manuscripts must include a data availability statement. This statement should provide the following information, where applicable:

- Accession codes, unique identifiers, or web links for publicly available datasets
- A description of any restrictions on data availability
- For clinical datasets or third party data, please ensure that the statement adheres to our policy

All data are available within the Article, Supplementary Information or Source Data file. Source data are provided with this paper.

Human research participants

Policy information about studies involving human research participants and Sex and Gender in Research.

Reporting on sex and gender	No
Population characteristics	No
Recruitment	No
Ethics oversight	No

Note that full information on the approval of the study protocol must also be provided in the manuscript.

Field-specific reporting

Please select the one below that is the best fit for your research. If you are not sure, read the appropriate sections before making your selection.

☒ Life sciences ☐ Behavioural & social sciences ☐ Ecological, evolutionary & environmental sciences

For a reference copy of the document with all sections, see nature.com/documents/nr-reporting-summary-flat.pdf

Life sciences study design

All studies must disclose on these points even when the disclosure is negative.

Sample size	All the experiments were performed using sample sizes based on standard protocols in the field and experimental experience."Inhibiting both proline biosynthesis and lipogenesis synergistically suppresses tumor growth. "J Exp Med,2020.
Data exclusions	No data was excluded from the study.
Replication	All biological experiments were carried out under clearly defined and standard conditions and were repeated at least twice whenever possible. All replication attempts were successful.
Randomization	Mice were randomly divided into different groups.For experiments other than mice experiments,samples were also randomly allocated into experimental groups.
Blinding	Investigators were blinded to group allocation during data collection and/or analysis.

Reporting for specific materials, systems and methods

We require information from authors about some types of materials, experimental systems and methods used in many studies. Here, indicate whether each material, system or method listed is relevant to your study. If you are not sure if a list item applies to your research, read the appropriate section before selecting a response.

Materials & experimental systems

n/a	Involved in the study
☐	☒ Antibodies
☐	☒ Eukaryotic cell lines
☒	☐ Palaeontology and archaeology
☐	☒ Animals and other organisms
☒	☐ Clinical data
☒	☐ Dual use research of concern

Methods

n/a	Involved in the study
☒	☐ ChIP-seq
☐	☒ Flow cytometry
☒	☐ MRI-based neuroimaging

Antibodies

Antibodies used

Second antibodies: the goat anti-rabbit IgG/HRP (Cat #ZDR-5306, 1:5,000 dilution) and the goat anti-mouse IgG/HRP (Cat#ZDR-5307, 1:5,000 dilution) secondary antibodies were obtained from ZSGB-Bio.
All antibodies used in this study, including name, supplier, catalogue number, species, reactivity, application, dilution and clone name are stated here in order:
β-Actin (Proteintech;60008-1-Ig;mouse; Human,Mouse,Rat,Plant,Zebrafish;FC, IHC, WB, ELISA;1:5000;7D2C10),
GAPDH (Proteintech;60004-1-lg;mouse;Human,Mouse,Rat,Yeast,Plant,Zebrafish;FC, IF, IP, WB, ELISA;1:5000;1E6D9),
ACC1 (Proteintech;21923-1-AP;Rabbit;Human,Rat,Mouse;FC, IF, IHC, IP, WB, ELISA;1:2000;none),
PRPS1 (Proteintech;15549-1-AP;Rabbit;Human,Mouse,Rat;IF,IHC,IP,WB,ELISA;1:5000;none),
PRPS2 (Proteintech;27024-1-AP;Rabbit;Human, Mouse;WB, ELISA;1:5000;none),
TKT (Proteintech;11039-1-AP;Rabbit;Human, Mouse, Rat;Human, Mouse, Rat;FC, IF, IHC, IP, WB, ELISA;1:5000;none),
PARP-1 (Proteintech;13371-1-AP;Rabbit;Human, Mouse, Rat;FC, IF, IHC, IP, WB, ELISA;1:1000;none),
Caspase-3 (Proteintech;19677-1-AP;Rabbit;Human, Mouse, Rat;FC, IF, IHC, IP, WB,ELISA;1:1000;none),
DHODH (Proteintech;14877-1-AP;Rabbit;Human, Mouse, Rat; IF, IHC, IP, WB, ELISA;1:1000;none),
Lamin B (Proteintech;12987-1-AP;Rabbit;Human, Mouse, Rat; FC, IF, IHC, IP, WB, ELISA;1:1000;none),
GFP (Proteintech;50430-2-AP;Rabbit; GFP-tag; IF, IP, WB, ELISA;1:5000;none),
α-Tubulin (Proteintech;11224-1-AP;Rabbit;Human, Mouse, Rat;FC, IF, IHC, IP, WB, ELISA;1:5000;none),
FLAG (Sigma;F1804;Mouse;Flag-tag;IF, IHC, IP, WB;1:5000;none) ,
pACC1-Ser 79 (CST;3661; Rabbit;Human, Mouse,Monkey,Rat;IHC, IP, WB;1:1000;none),
anti-pAMPK-Thr172(CST;2535;Rabbit;Human, Mouse,Monkey,Rat;IHC, IP, WB;1:1000;none),
and anti-AMPK (CST;5831;Rabbit;Human, Mouse,Monkey,Rat;IP, WB;1:1000;none)

Validation

PRPS1 and PRPS2 antibodies validated through detection of band at predicted molecular weight which was decreased in siRNA silencing cells. TKT antibody validated by detection of single band at predicted molecular weight which was decreased in knockout cells. FLAG antibody validated by detection of lentiviral overexpression of targets of the appropriate molecular weight. All antibodies validated by multiple citations and detailed information could be found on the website from manufactures as listed below.
Goat anti-rabbit IgG/HRP (ZSGB-Bio, Cat #ZDR-5306, http://www.zsbio.com/product/ZDR-5306)
Goat anti-mouse IgG/HRP (ZSGB-Bio, Cat #ZDR-5307, http://www.zsbio.com/product/ZDR-5307)
β-Actin (Proteintech, Cat#60008-1, http://www.ptgcn.com/products/ACTB-Antibody-60008-1-Ig.htm)
GAPDH (Proteintech, Cat# 60004-1-lg, http://www.ptgcn.com/products/GAPDH-Antibody-60004-1-Ig.htm)
ACC1 (Proteintech, Cat#21923-1-AP, http://www.ptgcn.com/products/GAPDH-Antibody-60004-1-Ig.htm)
PRPS1 (Proteintech, Cat#15549-1-AP, https://www.ptgcn.com/products/PRPS1-Antibody-15549-1-AP.htm)
PRPS2 (Proteintech, Cat#27024-1-AP, https://www.ptgcn.com/products/PRPS2-Antibody-27024-1-AP.htm)
TKT (Proteintech, Cat#11039-1-AP, https://www.ptgcn.com/products/TKT-Antibody-11039-1-AP.htm)
PARP-1 (Proteintech, Cat#13371-1-AP, https://www.ptgcn.com/products/PARP1-Antibody-13371-1-AP.htm)
Caspase-3 (Proteintech, Cat#19677-1-AP, https://www.ptgcn.com/products/CASP3-Antibody-19677-1-AP.htm)
DHODH (Proteintech, Cat#14877-1-AP, https://www.ptgcn.com/products/DHODH-Antibody-14877-1-AP.htm)
Lamin B (Proteintech, Cat#12987-1-AP, https://www.ptgcn.com/products/LMNB1-Antibody-12987-1-AP.htm)
GFP (Proteintech, Cat#50430-2-AP, https://www.ptgcn.com/products/eGFP-Antibody-50430-2-AP.htm)
α-Tubulin (Proteintech, Cat#11224-1-AP, https://www.ptgcn.com/products/TUBA1B-Antibody-11224-1-AP.htm)
FLAG (Sigma, Cat#F1804, https://www.sigmaaldrich.cn/CN/zh/substance/monoclonalantiflagm2antibodyproducedinmouse1234598765?context=product)
pACC1 (CST, Cat#3661S, https://www.cellsignal.cn/products/primary-antibodies/phospho-acetyl-coa-carboxylase-ser79-antibody/3661?site-search-type=Products&N=4294956287&Ntt=3661&fromPage=plp&_requestid=886390)
pAMPK (CST, Cat#2535, https://www.cellsignal.cn/products/primary-antibodies/phospho-ampka-thr172-40h9-rabbit-mab/2535?site-search-type=Products&N=4294956287&Ntt=2535&fromPage=plp&_requestid=2710695)
AMPK (CST, Cat#5831, https://www.cellsignal.cn/products/primary-antibodies/ampka-d5a2-rabbit-mab/5831?site-search-type=Products&N=4294956287&Ntt=5831&fromPage=plp&_requestid=2710765)

Eukaryotic cell lines

Policy information about cell lines and Sex and Gender in Research

Cell line source(s)

HeLa, MDA-MB-231, and HEK293T cell lines were obtained from ATCC. Primary embryonic fibroblasts (MEFs) were prepared from E13.5 wild type C57BL/6 embryos.

Authentication

Authentication was not performed as HeLa, MDA-MB-231, and HEK293T cell lines were not listed in the commonly misidentified lines.

Mycoplasma contamination	All cell lines tested negative for mycoplasma contamination.
Commonly misidentified lines (See ICLAC register)	No misidentified lines were used in this study.

Animals and other research organisms

Policy information about studies involving animals; ARRIVE guidelines recommended for reporting animal research, and Sex and Gender in Research

Laboratory animals	Male C57BL6/J mice purchased from Charles River, aged 6-8 weeks and female BALB/c nude mice purchased from Charles River, aged 5 weeks.
Wild animals	This study did not involve wild animals.
Reporting on sex	This study did not involve reporting on sex.
Field-collected samples	This study did not involve field-collected samples.
Ethics oversight	All animal protocols was approved by the IACUC at Capital Medical University in accordance with the principles and procedures outlined in the National Institutes of Health Guide for the Care and Use of Laboratory Animals.

Note that full information on the approval of the study protocol must also be provided in the manuscript.

Flow Cytometry

Plots

Confirm that:

☒ The axis labels state the marker and fluorochrome used (e.g. CD4-FITC).

☒ The axis scales are clearly visible. Include numbers along axes only for bottom left plot of group (a 'group' is an analysis of identical markers).

☒ All plots are contour plots with outliers or pseudocolor plots.

☒ A numerical value for number of cells or percentage (with statistics) is provided.

Methodology

Sample preparation	Cells were plated into a 12-well plate at 50000 cells per well. After treatment, all cells (including the upper floating dead cells) were collected and stained with 7AAD/Annexin V for 10 min at room temperature.
Instrument	Muse Cell Analyzer (Luminex Cooperation)
Software	FlowJo 7.6.
Cell population abundance	At least 5,000 cells were counted and analyzed.
Gating strategy	Gating strategy was presented in Supplementary Fig.3e, 4f, and 5f.

☒ Tick this box to confirm that a figure exemplifying the gating strategy is provided in the Supplementary Information.
